# Perioperative Blood Pressure Control in Carotid Artery Stenosis Patients With Carotid Angioplasty Stenting: A Retrospective Analysis of 173 Cases

**DOI:** 10.3389/fneur.2020.567623

**Published:** 2020-10-30

**Authors:** Longlong Zheng, Jiang Li, Haixiao Liu, Hao Guo, Lei Zhao, Hao Bai, Zhongjun Yan, Yan Qu

**Affiliations:** Department of Neurosurgery, Tangdu Hospital, The Fourth Military Medical University, Xi'an, China

**Keywords:** cerebral infarction, intracranial hemorrhage, carotid angioplasty stenting, carotid artery stenosis, blood pressure control

## Abstract

**Background:** Carotid angioplasty stenting (CAS) is a currently widely used surgical treatment of carotid artery stenosis. However, the influences of the perioperative blood pressure (BP) on patients' prognosis remain unclear.

**Objective:** The present study was designed to explore the effects of different perioperative BP control strategies on CAS patients' prognosis.

**Methods:** One hundred seventy-three consecutive patients admitted between January 2016 and April 2019 were reviewed retrospectively. The outcomes of patients with different systolic BP (<120, 120–130, and >130 mmHg) before CAS and within 24 h after CAS were compared. The primary outcomes were the incidence of secondary cerebral infarction (CI) and intracranial hemorrhage (ICH) after CAS. The secondary outcome was the incidence of unfavorable discharge and in-hospital death. The unfavorable discharge was defined as modified Rankin Scale (mRS) score 3–5 at discharge.

**Results:** There was no significant difference between the incidences of ICH (*P* = 0.803) and CI (*P* = 0.410) in patients with different BP before CAS. The patients with post-CAS BP values of >130 mmHg had a 37.67-fold increased risk (95% CI: 6.79–209.01) of ICH compared with others, while no significant difference was observed on the incidence of CI (*P* = 0.174) among patients with different post-CAS BP values. The patients with post-CAS BP values of >130 mmHg also had a significantly higher incidence of unfavorable discharge (*P* = 0.002) and in-hospital death (*P* = 0.001) compared with others.

**Conclusion:** High BP (>130 mmHg) within 24 h after CAS significantly increases the risks of secondary cerebral hemorrhage, unfavorable discharge, and in-hospital death. Thus, the BP should be controlled below 130 mmHg in the first 24 h after CAS.

## Introduction

Stroke has become the leading cause of death worldwide (1–3). Even worse, the absolute numbers of strokes will continue to increase worldwide for a long time along with the increasing of the aging population and the high prevalence of smoking and hypertension (4, 5), and most importantly due to the increasing incidence of stroke in younger persons aged <65 years (6, 7). In addition, the proportion of ischemic strokes is increasing in many developing countries such as China (4, 8). Currently, about 85% of strokes are cerebral ischemia, which is characterized by a sudden loss of neurological function due to insufficient blood supply to the brain (9).

Carotid artery stenosis is a well-documented risk factor for cerebrovascular diseases such as acute ischemic stroke or transient ischemic attack (10). It is reported that atherosclerotic disease of the extracranial internal carotid artery is responsible for 20–25% of ischemic strokes (11). The evidence of the optimal treatment strategies for different patients is still insufficient (10, 12). For asymptomatic carotid artery stenosis patients with stenosis <70%, medical therapy and control of risk factors are the currently recommended treatment strategies. However, for symptomatic patients with stenosis of more than 50% or asymptomatic patients with stenosis of more than 70%, surgical treatment could be considered (13–15).

Current surgical treatments for carotid stenosis include carotid endarterectomy (CEA) and carotid angioplasty stenting (CAS) (16). Although CEA has been established as the gold standard treatment for symptomatic severe carotid stenosis, CAS, as a less-invasive procedure, has been performed as an alternative to CEA (17, 18). Breakthroughs have been made since the treatment of endovascular recanalization for the symptomatic internal carotid artery stenosis patients in the subacute to chronic stage was first reported (19). Compared with CEA, CAS has advantages in the treatment of chronic carotid artery stenosis in some clinical situations (12, 20–23). For example, CAS patients can recover quickly and have shorter hospital stays. Besides, CAS can be performed on patients with severe stenosis of the internal carotid artery. Moreover, tandem lesions can be treated simultaneously.

Cerebral hyperperfusion syndrome (CHS), which could further lead to cerebral swelling or intracranial hemorrhage (ICH), occurs on 1.1–6.8% of intracranial arterial stenosis patients after CAS (24–26). The pathophysiology of CHS is characterized by the increase of cerebral blood flow due to the dysregulation of the cerebral vascular system and hypertension (27). Therefore, controlling post-CAS blood pressure (BP) at a low level could reduce the risk of CHS and ICH (28). However, excessive lowering of BP may aggravate the cerebral ischemia. Currently, the standard of BP control after CAS is still controversial.

Thus we conducted this retrospective study of 173 CAS patients at Tangdu Hospital from January 2016 to April 2019 to explore the effect of different perioperative BP control strategies on the risk of post-CAS ICH, cerebral infarction (CI), and prognosis of CAS patients.

## Materials and Methods

### Study Design and Population

The present study was designed to compare the incidence rate of CI, ICH, unfavorable discharge [modified Rankin Scale (mRS) 3–5], and in-hospital death in carotid artery stenosis patients with different perioperative BP after CAS. This study was approved by the Biological and Medical Ethics Committee of Tangdu Hospital (No. TDLL-20181205) and performed in Tangdu Hospital strictly following the Declaration of Helsinki (29). The medical records of all patients who underwent CAS at Tangdu Hospital between January 2016 and April 2019 were retrospectively reviewed.

Then, the participants were selected according to the following criteria.

### Inclusion Criteria

1. 18–80 years old.
2. Symptomatic patients with stenosis more than 50% or asymptomatic patients with stenosis more than 70% (demonstrated by digital subtraction angiography).
3. Without newly emerging ischemic stroke within 2 weeks.
4. Underwent CAS during the hospitalization.

### Exclusion Criteria

1. Definite contraindications against BP control (such as renal failure and hypovolemic shock).
2. A tendency of severe bleeding (such as a peptic ulcer or gastrointestinal bleeding).
3. A history of CEA or occlusion after stenting.
4. A history of ICH within 30 days.
5. Concurrent serious severe heart or lung dysfunction.

### Treatment

All patients obtained standard treatment in accordance with the recommendations of the American Heart Association/American Stroke Association (AHA/ASA). Medical histories were recorded after admission in a timely manner. Neurological status was documented on admission using the National Institutes of Health Stroke Scale (NIHSS) by certified neurologists. Routine radiological examination and blood tests were performed during the perioperative period. The strategies of BP control were specified based on the physician's judgment of the clinical status of patients.

### Surgical Procedures

All of the surgeries were performed by a well-trained vascular surgery team with more than 10 years of experience. All patients received antiplatelet medication (aspirin 100 mg/day and clopidogrel 75 mg/day) for at least 5 days before the surgery, which were performed under local anesthesia. A dose of 3,000 IU heparin was given by intravenous bolus injection intraoperatively to each patient. Cerebral angiography was performed before CAS to measure the stenosis degree according to the North American Symptomatic Carotid Endarterectomy Trial (NASCET) criteria. A protective distal filter device (EV3) was carefully deployed in the normal vessel distal to the stenosis. A balloon with a diameter of 4–6 mm was performed for pre- or post-dilation if needed. Then, a self-expanding stent was deployed over the lesion. Computed tomography (CT) or magnetic resonance imaging (MRI) was performed both before and after the surgery to assess ICH and CI.

### Data Collection and Outcomes Evaluation

General characteristics (such as sex, age, etc.) were collected from the patient information management department of our hospital. Past medical history (smoking, alcohol, hypertension, coronary artery disease, hyperlipidemia, and diabetes), degree of stenosis, and contralateral lesions were obtained from medical documentation and cerebral angiography report in our hospital. The preoperative severity of disease was evaluated by the preoperative NIHSS score, which was also collected from medical documentation. The baseline BP values were obtained as the mean values of three random systolic BP (SBP) in the last 24 h before CAS, and the post-CAS BP levels were acquired as the mean values of every hourly SBP in the first 24 h after CAS. If ICH or CI occurs within 24 h post-CAS, the subsequent BP values would be excluded from the analysis. All the BP records were collected from medical documentation. The outcomes of patients with different systolic BP (<120 mmHg, 120–130 mmHg, and >130 mmHg) before CAS and within 24 h after CAS were compared.

The primary outcomes were the incidence rate of ICH and CI after CAS during the hospitalization, which were assessed by CT or MRI scan. ICH was defined as punctate or confluent hyper-densities consistent with blood within the parenchyma of the cerebral hemispheres or within the subarachnoid space as demonstrated on CT imaging (30). CI was defined as episode of neurological dysfunction caused by focal cerebral based on CT or MRI (31). Spotty CI was ruled out, and carotid ultrasound was performed post procedure to assess thrombosis or occlusion of the carotid artery. All assessments were finished separately by two investigators after carefully comparing the cerebral imaging before and after CAS. The secondary outcomes were in-hospital death and unfavorable discharge, which was defined as mRS score 3–5 at discharge.

### Statistical Analysis

Statistical calculations were performed using SPSS version 23.0 (IBM, Armonk, USA). The chi-squared tests and Fisher's exact tests were used to analyze the comparison of categorical data, which were displayed as percentages. Wilcoxon signed-rank tests were used to analyze the comparison of continuous data, which were presented as median scores with interquartile ranges because of non-normal data distributions. Multivariate logistic regression analysis was used to detect the risk factors of ICH, CI, unfavorable discharge, and in-hospital death. Statistical test results were recognized as significant when the *P-*value was < 0.05.

## Results

### Patient Numbers

Two hundred eighty-eight consecutive patients with carotid artery stenosis who underwent CAS admitted to our hospital between January 2016 and April 2019 were retrospectively reviewed. Among them, 115 patients were excluded due to the lack of clinical or cerebral imaging data; then, a total of 173 patients met the inclusion criteria and were enrolled in this study.

### General Characteristics

Among the 173 included patients, 140 were male and 33 were female. The age of the patients ranged from 39 to 81, and the average age was 63.2 ± 9.0. The average degree of stenosis was 80.3 ± 8.3%. Among the 115 excluded patients, 97 were male and 18 were female. The age of the patients ranged from 42 to 80, and the average age was 63.7 ± 7.7. The average degree of stenosis was 78.5 ± 8.5%. There was no significant difference in the demographic and baseline characteristics among the BP groups divided according to the post-CAS systolic BP levels (Table 1).

**Table 1:** General characteristics in each group.

**Characteristics**	**Total**	**<120 mmHg**	**120–130 mmHg**	**>130 mmHg**	***P*-value**
Gender (female)	33/173	17.4% (20/115)	15.0% (6/40)	38.9% (7/18)	0.097
Age (years)		62.9 ± 8.7	63.2 ± 9.0	65.2 ± 11.1	0.749
Grade of hypertension		2 (0–3)	3 (2–3)	2.5 (2–3)	0.154
Diabetes mellitus		20.0% (23/115)	17.5% (7/40)	38.9% (7/18)	0.179
Coronary artery disease		13.0% (15/115)	12.5% (5/40)	11.1% (2/18)	1
Hyperlipidemia		27.0% (31/115)	32.5% (13/40)	38.9% (7/18)	0.502
Alcoholic		22.6% (26/115)	25.0% (10/40)	22.2% (4/18)	0.96
Smoking		48.7% (56/115)	45.0% (18/40)	33.3% (6/18)	0.501
Bilat lesions		9.6% (11/115)	17.5% (7/40)	22.2% (4/18)	0.159
Stenosis (NASCET, %)		79.1 ± 8.1%	81.4 ± 7.9%	85.4 ± 8.8%	0.006
Preoperative NIHSS score		0 (0–0)	0 (0–1)	0 (0–2.25)	0.426
Postoperative MRI		47.0% (54/115)	55.0% (22/40)	27.8% (5/18)	0.161
Spotty infarction		79.6% (43/54)	72.9% (16/22)	40.0% (2/5)	0.129

*NIHSS, National Institutes of Health Stroke Scale; MRI, Magnetic Resonance Imaging; NASCET, North American Symptomatic Carotid Endarterectomy Trial*.

## Outcome Assessment

After CAS, 14 (8.1%) patients suffered different types of ICH, which included 1 subarachnoid hemorrhage, 1 contralateral intracerebral microhemorrhage, 1 bilateral subdural hemorrhage, 1 posterior cerebral circulation hemorrhage, 6 intracerebral microhemorrhages, and 4 intracerebral hemorrhages. Nine (5.2%) patients suffered CI. Three (1.73%) patients died before discharge. Besides, unfavorable discharge occurred in six (3.47%) cases (Table 2). Among the 115 excluded patients, the incidence of ICH or CI cannot be identified due to the lack of clinical or cerebral imaging data. No one died or suffered unfavorable discharge.

**Table 2:** The incidence of post-CAS CI, ICH, unfavorable discharge, and in-hospital death in the patients with different baseline BP and post-CAS BP.

		**<120 mmHg**	**120–130 mmHg**	**>130 mmHg**	***P*-value**
Baseline BP	CI	0% (0/24)	7.9% (6/76)	4.1% (3/73)	0.410
	ICH	4.2% (1/24)	7.9% (6/76)	9.6% (7/73)	0.803
24 h BP	CI	3.5% (4/115)	7.5% (3/40)	11.1% (2/18)	0.174
	ICH	2.6% (3/115)	5.0% (2/40)	50% (9/18)	<0.0001
	Unfavorable discharge	1.7% (2/115)	0% (0/40)	22.2% (4/18)	0.002
	In-hospital death	0% (0/115)	0% (0/40)	16.7% (3/18)	0.001

*CI, Cerebral Infarction; ICH, Intracranial Hemorrhage*.

Among the three BP groups divided according to the baseline BP levels, there were no significant differences in the incidence of both CI (*P* = 0.410) and ICH (*P* = 0.803). However, among the three BP groups divided according to the post-CAS BP levels, there were significant differences on the incidence of ICH (*P* < 0.0001), unfavorable discharge (*P* = 0.002), and in-hospital death (*P* = 0.001), whereas there was no significant difference on the incidence of CI (*P* = 0.174) (Table 2).

Within different postoperative BP groups, the >130 mmHg group had a significantly higher incidence of ICH (*P* < 0.0001 vs. the <120 mmHg group, *P* < 0.0001 vs. the 120–130 mmHg group), unfavorable discharge (*P* = 0.003 vs. the <120 mmHg group, *P* = 0.007 vs. the 120–130 mmHg group), and in-hospital death (*P* = 0.002 vs. the <120 mmHg group, *P* = 0.026 vs. the 120–130 mmHg group) than that in the other two groups. However, there was no significant difference on the incidence of ICH (*P* = 0.604) and unfavorable discharge (*P* = 1) between the <120 mmHg group and the 120–130 mmHg group (Table 3).

**Table 3:** The difference in the incidence of post-CAS, ICH, unfavorable discharge, and in-hospital death between different post-CAS BP groups.

***P*-value**	**<120 mmHg vs. 120–130 mmHg**	**<120 mmHg vs. >130 mmHg**	**120 mm−130 mmHg vs. >130 mmHg**
ICH	0.604	<0.0001	<0.0001
Unfavorable discharge	1	0.003	0.007
In-hospital death	NA	0.002	0.026

*ICH, Intracranial Hemorrhage; NA, Not Applicable*.

After adjusting for confounding variables including age, baseline BP, the grade of hypertension, bilat lesions, degree of stenosis (NASCET), and preoperative NIHSS score, multivariate logistic regression analysis showed that the patients with post-CAS BP of >130 mmHg had a 37.67-fold increased risk (95% CI: 6.79–209.01) of post-CAS ICH compared with those with post-CAS BP of ≤ 130 mmHg (Table 4).

**Table 4:** Multivariate logistic regression of potential influentially factors on the post-CAS ICH.

		**OR (95% CI)**	***P*-value**
24 h BP (Reference is ≤ 130 mmHg)	>130 mmHg	37.67 (6.79–209.01)	<0.0001
Baseline BP		0.977 (0.91–1.05)	0.516
Age		0.938 (0.86–1.02)	0.130
Grade of hypertension		1.12 (0.59–2.12)	0.740
Bilat lesions (Reference is No)	Yes	1.67 (0.30–9.29)	0.560
Stenosis (NASCET)		1.194 (1.063–1.342)	0.003
Preoperative NIHSS score		0.54 (0.18–1.66)	0.282

*NIHSS, The National Institutes of Health Stroke Scale; NASCET, North American Symptomatic Carotid Endarterectomy Trial*.

Furthermore, the patients with post-CAS ICH had a 31.40-fold increased risk (95% CI: 5.13–192.59) of unfavorable discharge and a 26.33-fold increased risk (95% CI: 2.23–311.69) of in-hospital death, compared with others. Meanwhile, the patients with post-CAS CI had an 11.43-fold increased risk (95% CI: 1.78–73.30) of unfavorable discharge and a tendency of an increase of the risk (OR: 10.13, 95% CI: 0.83–123.75) of in-hospital death, compared with others (Table 5).

**Table 5:** Logistic regression of risk factors of unfavorable discharge and in-hospital death.

	**Unfavorable discharge**		**In-hospital death**	
	**OR (95% CI)**	***P*-value**	**OR (95% CI)**	***P*-value**
Hemorrhage				
NO	1.00		1.00	
YES	31.40 (5.13–192.59)	<0.0001	26.33 (2.23–311.69)	0.009
Infarction				
NO	1.00		1.00	
YES	11.43 (1.78–73.30)	0.01	10.13 (0.83–123.75)	0.07

**Figure 1:**
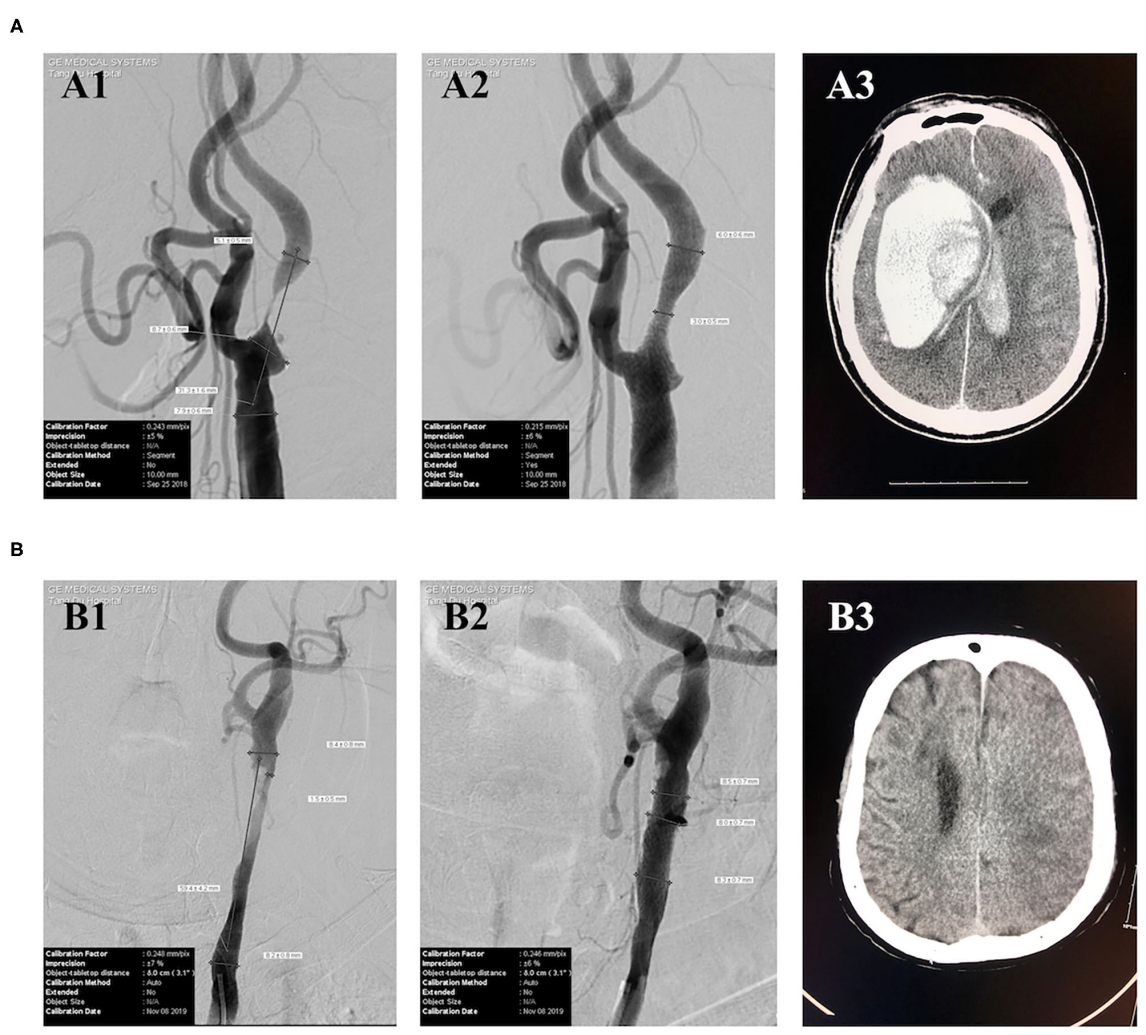
Typical cases of post-CAS ICH and CI. **(A)** Case #1 **(A1)** DSA showed a 99% stenosis in the proximal segment of the right internal carotid artery. **(A2)** DSA showed a 50% residual stenosis in the proximal segment of the right internal carotid artery after CAS. **(A3)** CT performed 25 h after CAS showed a huge right intracerebral hematoma. **(B)** Case #2 **(B1)** DSA showed an 82% stenosis in the left carotid artery. **(B2)** DSA showed a nearly 0% residual stenosis in the left carotid artery after CAS. **(B3)** CT scan performed 58 h after CAS showed a left massive CI.

Typical cases of post-CAS ICH and CI are shown in Figure 1. Case #1 was a patient with a 99% stenosis in the proximal segment of the right internal carotid artery. The baseline BP, immediate post-CAS BP, and average BP within 24 h after CAS were 125/75, 123/83, and 156/82 mmHg, respectively. This patient had symptoms of headache, nausea, and vomiting after CAS. The CT scan performed 25 h after CAS showed a huge right intracerebral hematoma (Figure 1A3), which was considered to be caused by poor post-CAS BP control and CHS. This patient eventually died in the hospital. Case #2 was a patient with an 82% stenosis in the left carotid artery. The baseline BP, immediate post-CAS BP, and average BP within 24 h after CAS were 125/61, 115/74, and 101/61 mmHg, respectively. The CT scan performed 58 h after CAS showed a massive left CI (Figure 1B3). Carotid ultrasound post-procedure excluded thrombosis or occlusion of the carotid artery. This patient eventually suffered unfavorable discharge.

## Discussion

The present study was designed to explore the effects of different perioperative BP control strategies on CAS patients' prognosis. We found that the post-CAS BP was significantly correlated with ICH, unfavorable discharge, and in-hospital death, while there was no significant association with CI in CAS patients, although baseline BP did not appear to have an effect on the incidence of both CI and ICH. The patients with post-CAS BP values of >130 mmHg had a 37.67-fold increased risk of ICH compared with others, and patients who suffered ICH had a 31.40-fold increased risk of unfavorable discharge and 26.33-fold increased risk of in-hospital death. These results suggest that the systolic pressure within 24 h after CAS should be controlled below 130 mmHg to avoid post-CAS ICH.

CAS has been demonstrated to be valuable in the treatment of chronic carotid artery stenosis. Post-CAS ICH is characterized with early occurrence, high disability, and mortality. As the most common cause, the mechanisms of post-CAS CHS might include: (1) the failure of vessels' autoregulatory mechanisms during the sudden increase of cerebral blood flow after revascularization in long-standing hypoperfused tissues, (2) the disturbance of baroreflex secondary to revascularization, and (3) the disturbance of the trigeminovascular system (32). Thus, maintaining hemodynamic stability may be an important way to prevent post-CAS CHS and ICH. However, most of the previous literature about CHS and ICH was concentrated on the difference of post-surgery ICH incidence between CAS and CEA (30, 33–35). Some other studies were focused on the predictive risk factors of CHS and ICH after CAS (36–38). Besides, as ischemic post-CAS stroke, the mechanisms of CI can be classified as (1) carotid-embolic, (2) hemodynamic, (3) thrombosis or occlusion of the carotid artery, (4) hyperperfusion, (5) cardio-embolic, (6) multiple, or (7) undetermined (39). A previous study showed that hemodynamic disturbance was an important mechanism, and careful attention to BP control could lower the incidence of ischemic post-CAS stroke (31). There is still limited clinical evidence on how to prevent ICH and CI after CAS, which is one of the primary issues in the management of patients and can directly improve the patient's prognosis.

In 1999, a literature first reported ICH after CAS and pointed out that attention should be paid to perioperative BP (40). In 2017, A meta-analysis showed that periprocedural hypertension was the most common risk factor of CHS and ICH (32). Another literature (39) proved that careful attention to BP control could lower the incidence of procedural stroke. In addition, previous studies have reached a common conclusion that post-CAS BP should be controlled below a certain level (41–44). However, it is still unclear whether lower BPs increase the risk of post-CAS CI. Although the present study showed that difference between the incidence of CI in each post-surgery BP group had no statistical significance, we provided a more specific strategy of BP control after CAS than the previous studies.

This study has several limitations. At first, the incidence of post-CAS ICH in this study was 8.1%, which was much higher than the incidence range of 0.36–4.5% reported in previous studies (26, 38). The incidence may be enlarged by two reasons. One is the selection bias inherent in a retrospective study, which is characterized by excluding 115 patients in whom no one died or suffered unfavorable discharge in this study, despite the lack of clinical or cerebral imaging data. Another is that we have included six intracerebral microhemorrhage patients who suffered no CHS symptoms in the analysis, while most of the previous studies focused on ICH in the context of CHS. Besides, the 5.2% incidence of CI is similar to the 4.3% of a previous study (30). Although we have excluded spotty CI to be directly related to the operation by MRI and assessed thrombosis or occlusion of the carotid artery by carotid ultrasound post-procedure, it is difficult to identify whether post-CAS CI was caused by carotid-embolic or hemodynamics. The reason we did not find significant association between post-CAS BP and CI may be the lack of essential cerebral imaging data and the small sample size of retrospective study. Last, the characteristics of a single-center study and the lack of randomization due to the retrospective design may not make the results generalizable. Thus, prospective multi-center randomized controlled clinical trials with a larger sample size and adequately pre-defined radiological examination protocol are needed.

In conclusion, the baseline systolic BP has no significant influence on post-CAS ICH and CI, while the BP within 24 h after CAS is closely related to the post-CAS ICH and patients' prognosis and should be controlled lower than 130 mmHg.

## Data Availability Statement

The original contributions presented in the study are included in the article/supplementary materials, further inquiries can be directed to the corresponding author/s.

## Ethics Statement

This study was approved by the biological and medical ethics committee of Tangdu Hospital (No. TDLL-20181205).

## Author Contributions

All authors of this work met ICMJE criteria for authorship and made substantial contributions to the conception and design, acquisition of data, analysis and interpretation of data, drafting, critical revising, and final approval of this manuscript.

## Conflict of Interest

The authors declare that the research was conducted in the absence of any commercial or financial relationships that could be construed as a potential conflict of interest.
